# DNA damage response coregulator *ddrR* affects many cellular pathways and processes in *Acinetobacter baumannii* 17978

**DOI:** 10.3389/fcimb.2023.1324091

**Published:** 2024-01-11

**Authors:** Deborah Cook, Mollee D. Flannigan, Julia H. Chariker, Janelle M. Hare

**Affiliations:** ^1^ Department of Biology and Chemistry, Morehead State University, Morehead, KY, United States; ^2^ Kentucky IDeA Networks of Biomedical Research Excellence (KY INBRE) Bioinformatics Core, University of Louisville, Louisville, KY, United States

**Keywords:** DNA damage response, *ddrR*, *Acinetobacter baumannii*, RNAseq, pathway analysis

## Abstract

**Introduction:**

*Acinetobacter baumannii* strain 17978 is an opportunistic pathogen possessing a DNA damage response (DDR) in which multiple error-prone polymerase genes are co-repressed by a UmuD homolog, UmuDAb, and the small *Acinetobacter*-specific protein DdrR. Additionally, these regulators coactivate nine other genes. We identified the DNA damage-inducible transcriptome for wildtype, *umuDAb*, and *recA* strains, and later established the *ddrR* DDR transcriptome. However, the ATCC 17978 reference genome had several assembly errors and lacked the 44 kb virulence locus, AbaAL44, that is present in the strain 17978 UN.

**Methods:**

For this project, we combined our earlier single-end read RNAseq data with the *ddrR* paired-end reads and aligned these data to the improved 17978 UN genome assembly that resembled our laboratory strain, 17978 JH.

**Results:**

New DESeq2 analyses verified previous differentially expressed genes (DEGs) but also found 339 genes in 17978 JH that were not annotated or physically present in the older genome assembly. Sixty-three were differentially expressed after DNA damage, and 182 had differential basal expression when comparing *umuDAb*, *ddrR*, or *recA* strains to wildtype, with 94 genes’ expression unchanged. This work identified and characterized the 55 gene DNA damage-repressible transcriptome, 98% of which required either *umuDAb* or *ddrR* for repression. Two-thirds of these DEGs required both regulators. We also identified 110 genes repressed only in the *ddrR* strain, ~50% of which were due to increased basal expression levels. Basal gene expression in the *ddrR* mutant was further dysregulated independent of the DDR. Over 800 genes were upregulated, and over 1200 genes were downregulated compared to wildtype expression. Half of *A. baumannii’s* essential genes were upregulated in the *ddrR* strain, including cell division genes, and two-thirds of these were downregulated in the *umuDAb* strain.

**Discussion:**

The *ddrR* mutant upregulated genes enriched in translation, RNA metabolism, protein metabolism, AA/FA/cell-structure synthesis, and transport, while downregulating genes enriched in quorum sensing, biofilm production, secretion systems, pilus production, cell adhesion, and aromatics and chlorine degradation. Our data underscore the need for accurate and appropriately matched genome assemblies and indicate that *ddrR* affects approximately 60% of the genome, rendering it a potential target for *Acinetobacter baumannii* infection treatment.

## Introduction

*Acinetobacter baumannii* is a non-enteric, gram-negative opportunistic pathogen that commonly causes nosocomial infections and is challenging to treat due to many strains’ multiple antibiotic resistances (Peleg et al., 2008; Antunes et al., 2011; Visca et al., 2011). The emergence of carbapenem-resistant strains has prompted the World Health Organization to name *A. baumannii* as a top-priority critical pathogen (Tacconelli et al., 2018).

Several *Acinetobacter* species and strains are models for studying virulence-related processes such as antibiotic resistance, the DNA damage response (DDR), desiccation tolerance, and metabolism. Among the hundreds of *A. baumannii* strains that are currently studied, one of the oldest and most commonly used is *A. baumannii* ATCC 17978, isolated in 1951 from an infant with fatal meningitis and susceptible to most antibiotics (Smith et al., 2007). Antibiotic resistance in this pathogen is usually due to the gain or loss of genetic material, via plasmids or horizontal gene transfer (Dobrindt and Hacker, 2001; Antunes et al., 2014), but the DDR is mutagenic and can cause antibiotic resistance (Norton et al., 2013; Aranda et al., 2014; Durante-Mangoni et al., 2015; Zhu et al., 2019). The *A. baumannii* DDR that induces ~150 genes after DNA damage (Aranda et al., 2013; Hare et al., 2014) is unusual in not being regulated by LexA, as the genus does not encode a LexA homolog (Hare et al., 2012). Several features of this system and its coregulators UmuDAb and DdrR have been investigated in the non-pathogenic *A. baylyi* ADP1 and *A. baumannii* (Robinson et al., 2010; Hare et al., 2012; Aranda et al., 2013; Norton et al., 2013). Both species induce *umuDAb* and *ddrR* as part of their DDR (Hare et al., 2014). The products of this divergently transcribed gene cluster (Peterson et al., 2020) jointly repress the six plasmid- or prophage-based *umuD* and *umuC* genes (Hare et al., 2014) that have been acquired through horizontal gene transfer (Di Nocera et al., 2011; Hare et al., 2012). These *umuDC* genes encode subunits of the error-prone polymerase V (UmuD’_2_C) that conducts error-prone DNA repair and thus are under tight regulation. Polymerase V repair can lead to mutations in genes whose gain of function could potentially increase antibiotic resistance (Norton et al., 2013).

UmuDAb and DdrR constitute an unusual coregulatory system for DDR genes. *ddrR* encodes a ~9 kD protein that is unique to the *Acinetobacter* genus. When we investigated whether *umuDAb* and *ddrR* regulated all DNA damage-inducible genes in *Acinetobacter*, we conducted RNAseq analyses to identify the DDR-induced UmuDAb transcriptome in *A. baylyi* and *A. baumannii* (Hare et al., 2014), later extending the analysis to the DdrR transcriptome (Peterson et al., 2020). RNAseq is a cost-effective way to study the transcriptome of organisms, supplementing and often replacing microarray analysis. However, this powerful technique relies on an accurately sequenced and assembled genome to align cDNA reads. Unfortunately, the first and most used reference genome for ATCC 17978 (GCA_000015425.1) contains several errors introduced through bases lost during sequencing or by incorrectly combining contigs, notably the chromosome and one of the three plasmids, pAB3 (Smith et al., 2007; Weber et al., 2015). For example, the *lon* gene was annotated as two open reading frames (*A1S_1031* and *A1S_1030*), because a base was missed in a poly A or T region during sequencing (Ching et al., 2018). After sequencing our lab strain 17978 JH, we observed that the gene transcribed convergently with *ddrR* (*A1S_3662; dtpA* (Green et al., 2022) contained a 400 bp high-GC repeat region that was missing from the ATCC 17978 assembly (D. Cook, personal communication). That apparent assembly mistake caused a frameshift, so *dtpA* appears to extend through both genes’ observed transcription termination sites (Kröger et al., 2018). A recent *A. baumannii* study confirms that this region with the full-length *dtpA* gene, is conserved among *A. baumannii* strains AB5075, 19606, and 17978-mff (Green et al., 2022).

Several documented genomic differences between different laboratories’ ATCC 17978 strains have also complicated past studies. In 2018, Kröger et al. investigated the transcriptome of 17978 using the University of Alberta’s *A. baumannii* 17978-mff assembly, which contained the pAB3 plasmid, as their reference genome. However, the Kröger lab strain had lost the large plasmid pAB3 during transit and their transcriptome data only represented the chromosome. In another example, a 44 kb virulence locus, AbaAL44 (*Acinetobacter baumannii* accessory locus 44 kb), was identified as both present and absent in an admixture found in two different laboratory *A. baumannii* 17978 strains purchased from the American Type Culture Collection (ATCC) (Wijers et al., 2021). The AbaAL44 locus is present in sequenced strains 17978-mff, 17978 UN, and our strain 17978 JH but is absent from the original ATCC 17978 and 17978 VU strains, probably due to the locus’ loss during laboratory propagation (Wijers et al., 2021). Similar genomic variations between different laboratories’ cultures have been seen in *A. baumannii* 19606 (Artuso et al., 2022). These examples show how variable and constantly changing the *A. baumannii* genome is: only 14.8% to 16.5% of the genome is shared among 12 (Imperi et al., 2011) or 47 (Philippe et al., 2022) isolates, respectively.

Our RNAseq study of the 17978 JH DNA damage transcriptome used the ATCC 17978 reference genome for read alignments and CuffDiff analysis to find differentially expressed genes (DEGs) (Hare et al., 2014; Peterson et al., 2020). However, due to assembly errors and the absence of AbaAL44 from the reference genome, our analysis was incomplete and less accurate than desired. Fortunately, technological advancements have improved the quality and accuracy of genome assembly. This project’s goals were to: (i) extend our earlier investigation of the induced DDR to obtain the repressed DDR and its regulation by *ddrR* and *umuDAb*, and (ii) improve our gene discovery methods. We therefore aligned the RNAseq data to the more accurate 17978 UN genome assembly that matches our lab strain 17978 JH and updated our analysis pipeline to use DESeq2. That Bioconductor package uses a statistical model to estimate the mean and variance of the count data, which controls differences in sequencing parameters between samples (such as single or paired-end reads). This allowed the single-read data for 17978 JH, the *umuDAb* mutant, and the *recA* mutant (Hare et al., 2014) to be combined with and directly compared to paired-read data from the *ddrR* mutant (Peterson et al., 2020).

With these improved methods, we verified previously reported DDR genes and found new ones that had not been annotated or were absent from the ATCC 17978 genome assembly. We also compared basal expression levels and found genes and pathways whose expression was affected by mutating the regulators *umuDAb*, *ddrR*, and *recA*, independent of DNA damage treatment. This analysis showed that *ddrR* plays a much more significant role than simply co-regulating error-prone polymerases and the *ddrR*-*umuDAb* overlapping promoters. *ddrR* affects the expression of genes for quorum sensing, efflux pumps, pilus production, the TCA cycle, RNA transcription and translation, and other metabolic processes. This means it could be an effective therapeutic target to aid in treating *Acinetobacter baumannii* infections.

## Materials and methods

RNAseq reads from previously published work were used (Hare et al., 2014, Peterson et al., 2020). In those projects, the RNAseq analysis was to find DEGs after the cells experienced DNA damage caused by mitomycin C (MMC). The first RNAseq dataset included samples from three *Acinetobacter* strains: wildtype ATCC 17978 JH, and two single-mutant strains 17978 JH *recA*::*Km* (Aranda et al., 2011), and 17978 JH Δ*umuDAb*::*Km* (JH1600; (Hare et al., 2014)).

Sequencing reads were collected as 160 bp unpaired cDNA reads (Hare et al., 2014). The read clusters were normalized by multiplying each sample’s coverage by the total reads of the lower read-count sample divided by the respective sample’s total reads, and the induction ratio of reads was calculated between MMC-treated and untreated samples. The gene was induced if the induction ratio was greater than or equal to 2.0 and repressed if it was less than 0.5. No p-values or false discovery rate values were used (Hare et al., 2014). The second study consisted of a new RNAseq data set for a 17978 *ddrR*::Tn*LK* mutant strain (JH1700; (Peterson et al., 2020), with RNA processed as 75 bp paired-end cDNA reads. These data were normalized and analyzed separately from the data from the earlier study (Hare et al., 2014). Gene expression levels were calculated by FPKM (fragments per kilobase of transcript per million fragments mapped reads), then tested for differential gene expression with CuffDiff with a false discovery rate (FDR) threshold of q < 0.01 for genes that were repressed or induced greater than 2-fold according to FPKM (Peterson et al., 2020). Samples for both studies were collected by the same person, in the same make of test tubes, in the same shaking incubator in the same lab, in the same medium and volume at the same time, temperature, and aeration conditions, and RNA prepared with the same purification kit.

For this project, we first compared DEGs repressed after MMC treatment in the wildtype 17978 JH strain (WT) using data aligned to both the newer 17978 UN assembly GCA_019356215.1 (Wijers et al., 2021) and the earlier ATCC 17978 reference genome GCA_000015425.1 (for validation purposes). We used the final counts files, prepared by the KY INBRE Bioinformatics core, with the reads aligned to each genome using STAR aligner (Dobin et al., 2013). DESeq2 analysis was performed from the raw read counts. DESeq2 normalizes RNAseq experiment count data and finds DEGs between samples based on the negative binomial distribution (Love et al., 2014). For both MMC-treated and basal expression analyses, genes were considered differentially expressed if they had an adjusted p-value (q value) of < 0.05. The log2 fold change filter for values 1 < FC < -1 was not used in this analysis, to identify more biologically relevant differences in genes with low expression. Outliers were calculated in DESeq2 using Cook’s cutoff. Expression differences between WT and mutant strains were verified with RT-qPCR for several genes as performed previously (Hare et al., 2014; Peterson et al., 2020) and with new primers for additional genes (See **Supplementary Table S1**).

Pathway and gene enrichment analyses and images were created using the 17978 UN genome on the BioCyc website with our DESeq2 results overlaid in Cellular Overview (Paley and Karp, 2006) and enrichment scores calculated from the same data in the Omics Dashboard using padj < 0.05 as the threshold (Paley et al., 2017).

## Results

### 95 percent of induced genes were the same in the separate and merged RNASeq data sets, validating a merged analysis

We previously analyzed and published the *ddrR* transcriptome separately from the WT, *umuDAb* mutant, and *recA* mutant transcriptomes (Hare et al., 2014; Peterson et al., 2020). In this project, we tested whether combining the *ddrR* paired-end reads with the single reads of the other three strains into a single data set would alter the DESeq2 results for induced WT genes after MMC treatment. DESeq2 was applied both to the original counts file that contained only the reads for WT and the *umuDAb* and *recA* mutants, and to a counts file containing these strains’ data and those of the *ddrR* mutant. The combined mean for each gene was different due to the broader range of values in the *ddrR* data, but the overall results were very similar. Virtually all induced DEGs were the same in both analyses (170/177), with only two genes unique to the unmerged analysis and five genes that only appeared in the merged data set (**Supplementary Table S2**). All the results presented in this manuscript are from DESeq2 analysis of the merged data counts files.

### DNA damage-responsive genes identified after alignment to each reference genome

After alignment and DESeq2 analysis of the entire data set, induced or repressed DEGs were identified for each strain after MMC treatment. The DESeq2 summary results from each genome alignment and strain are in **Table 1**, and the complete data set is in **Supplementary Table S3**. The new (17978 UN) genome alignment found 339 genes in 17978 JH that were not annotated or physically present in the older (ATCC 17978) reference genome, 43 of which were differentially expressed after MMC treatment and 182 (181 in the *ddrR* mutant alone) that had differential basal expression compared untreated WT. **Supplementary Table S3** only includes the WT 17978 JH strain and the *umuDAb* and *ddrR* mutants because no genes were induced or repressed after MMC treatment in the *recA* mutant.

**Table 1:** Summary data for genes differentially expressed after MMC treatment.

adjusted p-value < 0.05		ATCC 17978	17978 UN
	Total Read Count	3877	3863
**Induced**	WT	143 (3.7%)	174 (4.5%)
Log_2_ fold change > 0 (up)	*umuDAb*	210 (5.4%)	223 (5.8%)
	*ddrR*	210 (5.4%)	238 (6.2%)
**Repressed**	WT	51 (1.3%)	54 (1.4%)
Log_2_ fold change < 0 (down)	*umuDAb*	9 (0.23%)	10 (0.26%)
	*ddrR*	118 (3%)	131 (3.4%)
Outliers (DESeq2 cutoff parameters used)	WT	1 (0.026%)	1 (0.026%)
	*umuDAb*	2 (0.052%)	1 (0.026%)
	*ddrR*	1 (0.026%)	1 (0.026%)

### Regulation of DNA damage-responsive genes by *ddrR* and *umuDAb*

**Figure 1:**
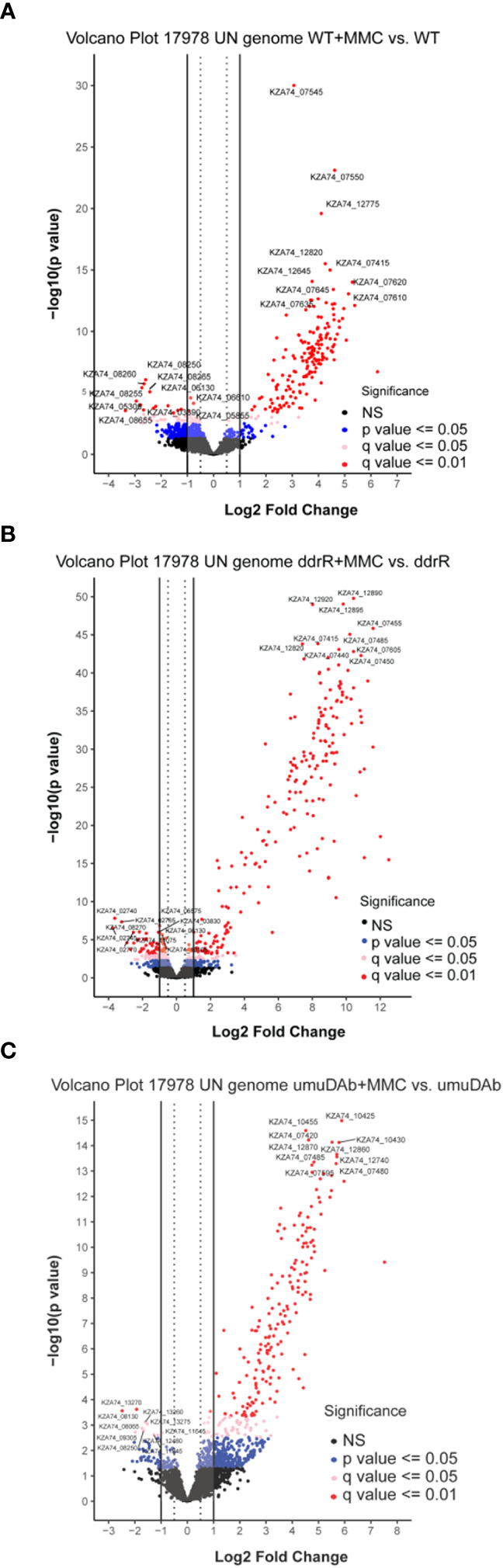
Global transcriptomic analysis of differentially expressed genes after MMC treatment for **(A)** wildtype (WT), **(B)** *ddrR*, and **(C)** *umuDAb* mutant strains of *A. baumannii* 17978 JH. Volcano plots show the log2 fold changes in gene expression where each dot represents one gene. Red and pink dots indicate significance at the q < 0.01 or q < 0.05 level. The log2 fold change is plotted on the x-axis, and the y-axis shows the log10 of the p value. The 10 genes with the greatest induction or repression are labelled.

**Figure 2:**
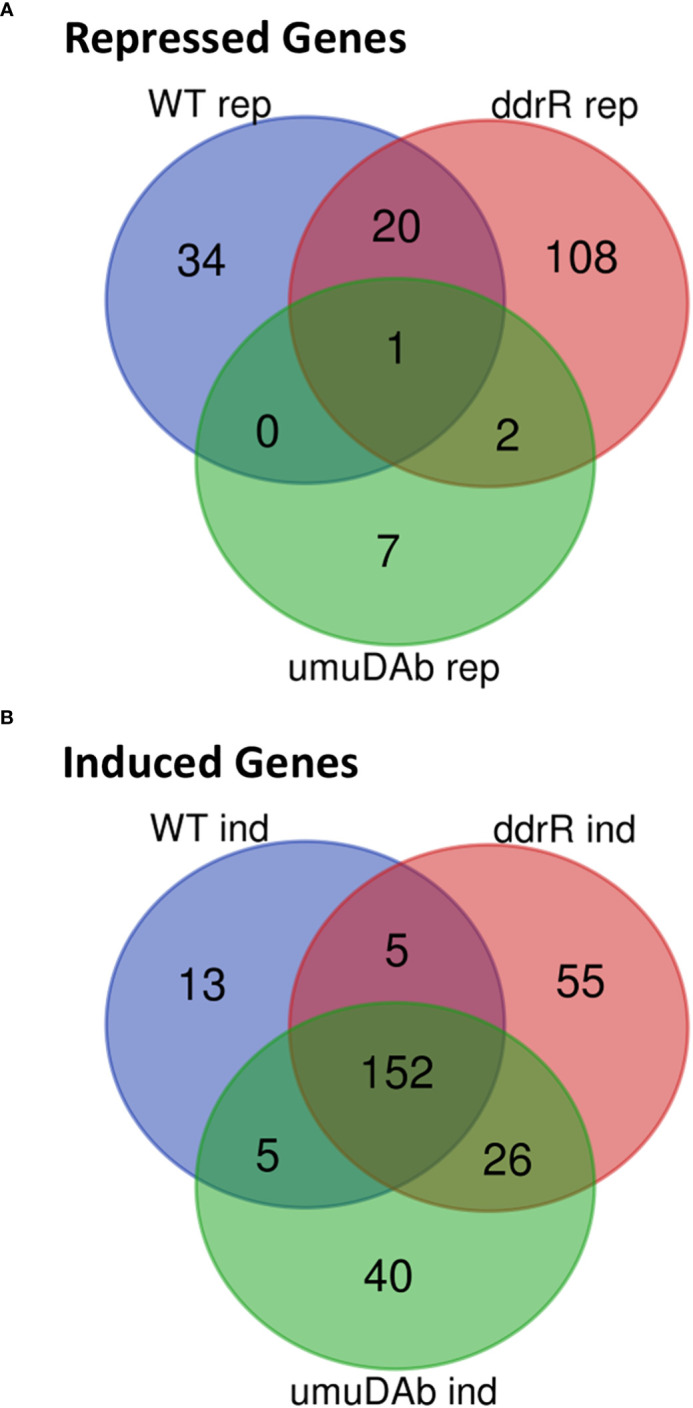
Venn diagram of differential gene expression, showing genes that were **(A)** repressed or **(B)** induced after MMC treatment (q < 0.05 for each) in wildtype (WT), *ddrR*, and *umuDAb* mutant strains of *A. baumannii* 17978 JH.

The WT DNA damage response resulted in 175 induced genes and 55 repressed genes (**Figures 1A**, **2B**). Interestingly, there were even more genes induced (238) and repressed (131) in the *ddrR* mutant (**Figure 1B**), including many of the same genes induced as WT (**Figure 2**). Lastly, in the *umuDAb* mutant, DNA damage resulted in 223 genes induced and 10 repressed (**Figures 1C**, **2**). Most of the induced genes reported here were previously published but used different analysis pipelines, fold change threshold, and genome assembly (Hare et al., 2014; Peterson et al., 2020) than this project. Additional findings from this study’s analysis are discussed in each pertinent section below.

#### umuDAb and ddrR were jointly needed to repress most genes repressed after DNA damage

To complement our understanding of genes induced by DNA damage treatment, we investigated genes that were repressed in WT cells after MMC treatment. The results for both the older and the new reference genomes were combined in **Table 2** to highlight DEG differences that directly resulted from the reference assembly used. Both genomes agreed for 43 genes of the 53 or 55 (using ATCC 17978 or 17978 UN, respectively) genes repressed in WT cells (**Table 2**). The categorization as being regulated by UmuDAb and DdrR, or only UmuDAb, differed for a minority (18) of the 43 genes repressed in both alignments. This could be due to the improved method of using q-values to assess significance rather than the simpler two-fold induction or repression ratio used previously, as well as improved annotations of entire genes rather than fragments of genes and newly annotated genes, including those in AbaAL44, causing changes in read depth for other genes. In the rest of our analysis of MMC-repressed genes, we considered only results from alignment to the 17978 UN genome.

**Table 2:** Genes repressed after DNA damage that are regulated by *ddrR* or *umuDAb*.

	Reads Aligned to ATCC 17978	*umuDAb*	*ddrR*	Reads Aligned to 17978_UN	*umuDAb*	*ddrR*
1	A1S_3278/KZA74_01040	NSC	NSC	KZA74_01040	NSC	NSC
2	A1S_3858/*yfbU*	NSC	NSC	KZA74_02675	NSC	NSC
3	A1S_2906/KZA74_02980	NSC	NSC	KZA74_02980	NSC	NSC
4	A1S_2834/*mscL*	NSC	NSC	KZA74_03335	NSC	NSC
5	A1S_2823/KZA74_03390	NSC	NSC	KZA74_03390	NSC	NSC
6	A1S_2820/KZA74_03405	NSC	NSC	KZA74_03405	NSC	NSC
7	A1S_2667/KZA74_04205	NSC	NSC	KZA74_04205	NSC	NSC
8	A1S_2629/KZA74_04435	NSC	NSC	KZA74_04435	NSC	NSC
9	A1S_2514/KZA74_05010	NSC	NSC	KZA74_05010	NSC	NSC
10	A1S_2510/KZA74_05040	NSC	R	KZA74_05040	NSC	R
11	A1S_2459/KZA74_05305	NSC	NSC	KZA74_05305	NSC	NSC
12	A1S_2458/KZA74_05310	NSC	NSC	KZA74_05310	NSC	NSC
13	A1S_2354/KZA74_05795	NSC	NSC	KZA74_05795	NSC	NSC
14	A1S_2342/KZA74_05855	R	R	KZA74_05855	NSC	R
15	A1S_2317/*rlpA*	NSC	NSC	KZA74_05975	NSC	R
16	A1S_2288/KZA74_06130	NSC	NSC	KZA74_06130	NSC	R
17	A1S_2266/KZA74_06250	NSC	R	KZA74_06250	NSC	R
18	A1S_2263/KZA74_06265	NSC	R	KZA74_06265	NSC	R
19	A1S_2241/KZA74_06380	NSC	R	KZA74_06380	NSC	NSC
20	A1S_2204/KZA 74_06585	NSC	R	KZA74_06585	NSC	R
21	A1S_2200/KZA74_06610	NSC	R	KZA74_06610	NSC	R
22	A1S_2192/KZA74_06650	NSC	R	KZA74_06650	NSC	NSC
23	A1S_1926/KZA74_08250	NSC	NSC	KZA74_08250	R	R
24	A1S_1925/*cydB*	NSC	NSC	KZA74_08260	NSC	R
25	A1S_1924/KZA74_08265	NSC	R	KZA74_08265	NSC	R
26	A1S_1845/*catA*	R	R	KZA74_08655	NSC	NSC
27	A1S_1844/*catC*	NSC	NSC	KZA74_08660	NSC	NSC
28	A1S_1843/KZA74_08665	NSC	R	KZA74_08665	NSC	NSC
29	A1S_1701/KZA74_09665	R	R	KZA74_09665	NSC	NSC
30	A1S_1700/KZA74_09670	NSC	R	KZA74_09670	NSC	NSC
31	A1S_1699/KZA74_09675	R	NSC	KZA74_09675	NSC	NSC
32	A1S_1679/KZA74_09805	NSC	NSC	KZA74_09805	NSC	NSC
33	A1S_1671/KZA74_09845	NSC	NSC	KZA74_09845	NSC	NSC
34	A1S_1620/KZA74_10105	NSC	NSC	KZA74_10105	NSC	R
35	A1S_1618/KZA74_10115	NSC	NSC	KZA74_10115	NSC	R
36	A1S_1548/KZA74_10625	NSC	NSC	KZA74_10625	NSC	R
37	A1S_1518/KZA74_10790	NSC	NSC	KZA74_10790	NSC	NSC
38	A1S_1498/KZA74_10910	NSC	NSC	KZA74_10910	NSC	NSC
39	A1S_1492/KZA74_10940	NSC	NSC	KZA74_10940	NSC	NSC
40	A1S_0771/KZA74_14830	NSC	R	KZA74_14830	NSC	NSC
41	A1S_0549/KZA74_15695	NSC	NSC	KZA74_15695	NSC	R
42	A1S_0548/KZA74_15700	NSC	NSC	KZA74_15700	NSC	R
43	A1S_0292/KZA74_16950	NSC	NSC	KZA74_16950	NSC	NSC
44	A1S_2487/KZA74_05150	NSC	R	KZA74_09360^Ŧ^	NSC	NSC
45	A1S_2468/KZA74_05255	NSC	R	KZA74_03465 ^Ŧ^	NSC	NSC
46	A1S_2428/KZA74_05450	NSC	NSC	KZA74_03595	NSC	NSC
47	A1S_3802*/KZA74_05730 A1S_3801	NSC	R	KZA74_03825	NSC	R
48	A1S_2264/KZA74_06260	NSC	R	KZA74_05865	NSC	R
49	A1S_2258/KZA74_06295	NSC	R	KZA74_06170	NSC	NSC
50	A1S_1702/KZA74_09660	NSC	R	KZA74_08255 ^Ŧ^	NSC	R
51	A1S_0965/KZA74_13855	NSC	R	KZA74_10080	NSC	NSC
52	A1S_0857/KZA74_14395	NSC	R	KZA74_10660	NSC	R
53	A1S_0839*/KZA74_14490 A1S_0838	NSC	R	KZA74_10905	NSC	NSC
54				KZA74_11345	NSC	R
55				KZA74_14620	NSC	NSC

NSC, No significant change after DNA damage, i.e., not repressed or induced.

R, Repressed after DNA damage.

Yellow cells, genes that were repressed in both reference genomes.

Gray cells, genes with regulation differences in mutants, depending on the reference genome alignment.

Ŧ, Genes that were newly annotated in 17978 UN relative to ATCC 17978.

*, Pairs of genes that were incorrectly annotated, as they are a single gene. Reading frame in bold was differentially expressed.

**Figure 3:**
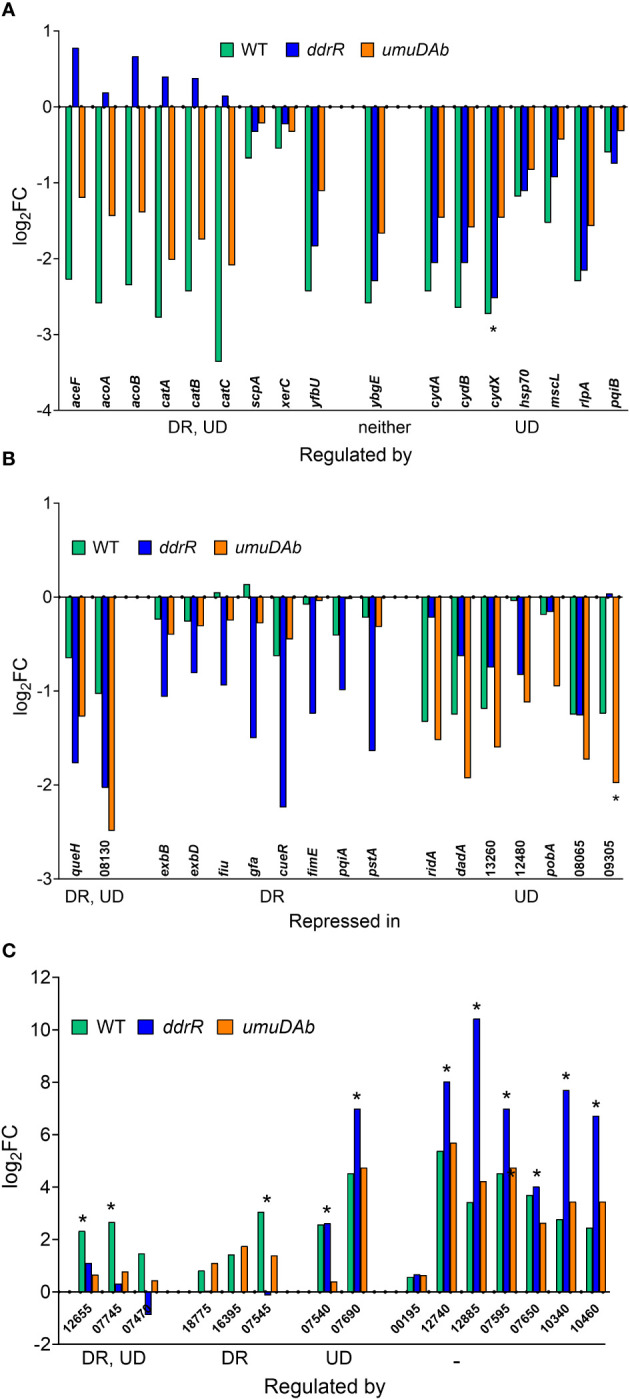
Genes responsive to MMC treatment in WT, *ddrR* and *umuDAb* mutant strains. RNASeq data of log_2_ fold changes is shown and categorized by response to MMC treatment, either repressed after treatment (panels A and B) or induced after treatment (panel C). **(A)** Genes that were repressed in the WT strain and whose repression was regulated by both *ddrR* and *umuDAb* (DR, UD), neither gene (-), or *umuDAb* (UD). **(B)** Genes that were not repressed in the WT strain but were repressed in the *ddrR* and/or *umuDAb* mutant strains. **(C)** Genes newly identified as induced after MMC treatment in this study. Shown are all eight induced genes regulated by either *ddrR* or *umuDAb*, or both genes, or neither gene (-). Unregulated genes include chromosomal gene KZA74_00195 and several CP genes (KZA74_12740 and 12885 from CP5, 07595 and 07650 from CP9, and 10340 and 10460 from CP14). Asterisks denote genes that had not been previously annotated in *A. baumannii* ATCC 17978. Numbers for gene names represent the KZA74 gene annotations.

Of the 55 genes that were repressed by MMC in the WT strain, 34 were no longer repressed in either the *ddrR* or *umuDAb* mutant (i.e., required each for repression) and 20 of the remaining 21 genes required only *umuDAb* for repression (**Figures 1**, **2A**). No genes required only *ddrR* for repression, and only one gene (*ybgE*, *KZA74_08250*) was repressed in WT and not regulated by either *ddrR* or *umuDAb* after MMC treatment (**Figure 3A**). Six normally repressed genes were not repressed in the *ddrR* mutant after DNA damage because they were already downregulated more than two-fold (repressed with no treatment) before MMC treatment (*catABC*, *acoAB*, and *aceF;* involved in catechol metabolism and acetoin catabolism). Seven other genes normally repressed after DNA damage were dysregulated in the absence of *ddrR*. They were expressed at levels more than two-fold higher than untreated WT and were not repressed after DNA damage (**Figure 3A**).

The AbaAL44 locus pathogenicity island possesses 44 genes (Wijers et al., 2021). After aligning our cDNA reads to the 17978 UN genome, six of these genes, *KZA74_9200 - 9210* and *KZA74_09245 - 09255* had no read coverage from our strains. Of the 38 genes for which we had read coverage, three were repressed after MMC treatment, each in a different strain: *ppk1* (*KZA74_09360*) in WT, *clsC* (*KZA74_09315*) in the *ddrR* mutant, and hypothetical *KZA74_09305* in the *umuDAb* mutant (**Supplementary Table S4**).

#### *ddrR* and *umuDAb* mutants repressed over 100 genes not DNA damage-repressed in wildtype

Surprisingly, multiple genes not repressed in WT cells were repressed in either the *ddrR* or *umuDAb* mutant strains in response to DNA damage. Two genes were repressed in both *umuDAb* and *ddrR* mutant strains after MMC treatment: a hypothetical gene *KZA74_08130* that was not annotated in the ATCC 17978 genome, and *KZA74_11845 queH*, epoxyqueuosine reductase (**Figure 3B**). Seven genes were repressed only in the *umuDAb* mutant: *pobA, dadA* homolog *KZA74_13270*, *ridA* homolog *KZA74_13275*, and four hypothetical genes (**Figure 3B**).

However, most (108) of the 117 genes were repressed solely in the *ddrR* mutant (**Supplementary Table S5**). Nearly half (49) of these genes repressed only in the *ddrR* mutant were repressed from dysregulated basal levels much higher than in WT. These included copper resistance genes *cueR, copA*, and *copB* (Williams et al., 2016; Williams et al., 2020). Interestingly, we found a highly upregulated 195 bp coding sequence that appeared to be a 5’ UTR of the *cydA* gene. Both *cydA* and *cydB* in the *cydABX* operon were modestly but not significantly upregulated and the *cydX* gene was severely downregulated in the *ddrR* mutant. After DNA damage, the 195 bp 5’ UTR region was repressed and the *cydB* gene was induced. This implies a possible regulatory effect of *ddrR* and the 5’ UTR region of *cydA*. The following pathways were enriched for these genes: amino acid, fatty acid/lipid, and cell structure synthesis, amino acid and amine degradation, translation, RNA metabolism, outer and plasma membrane components, and periplasm components.

In contrast, only ten of the genes repressed in the *ddrR* mutant had basal expression levels that were significantly *lower* than WT. Pathways enriched for these genes were involved in aromatic and chlorine degradation, aerobic respiration, adhesion, and pilus production, including the genes *exbB* and *exbD*, components of TonB dependent siderophore and vitamin B_12_ transport (Moeck and Coulton, 1998) and the fimbrial biogenesis gene, *fimE*, regulator of type I fimbriation in *Escherichia coli* (Blomfield et al., 1991). The remaining fifty genes were repressed from basal levels not significantly different from WT. None of these 108 genes were in a cryptic prophage (CP) and only one was on a plasmid, hypothetical (*KZA74_19035*) on pAB3.

#### Identification of 50 new DNA damage-inducible genes, several regulated by *umuDAb* and *ddrR*

The coregulators UmuDAb and DdrR that jointly repress the *umuD*-*umuC* error-prone polymerases before DNA damage, also are required to induce nine DDR genes (Peterson et al., 2020). We tested whether the new annotations in 17978 UN would reveal additional co-regulated genes that had either escaped annotation previously or were in the AbaAL44 region. Of the 175 genes induced in WT cells after MMC treatment (**Supplementary Table S2**), 50 were newly identified as induced, 40 were not annotated in the earlier reference genome and none were in AbaAL44 (**Figure 2A**, **Table 3**, **Supplementary Table S4**). All but three of these 50 DEGs were in cryptic prophages CP5, CP9, and CP14 (Di Nocera et al., 2011). One was in pAB3 (hypothetical gene *KZA74_18775*) and the other two were chromosomally encoded (hypothetical genes *KZA74_00195* and *KZA74_16395*, which was transcribed convergently to the induced *gst A1S_0408* (**Figure 3C**).

**Table 3:** Newly identified genes induced in WT after DNA damage.

Induced in:	ENSEMBL GENE	17978 A1S_ locus	*ddrR* basal expression
WT *ddrR umuDAb*	KZA74_00195	A1S_3441	NSD
WT *ddrR umuDAb*	KZA74_07415	NA	downreg
WT *ddrR umuDAb*	KZA74_07420	NA	downreg
WT *ddrR umuDAb*	KZA74_07425	NA	downreg
WT *ddrR umuDAb*	KZA74_07450	NA	downreg
WT	KZA74_07470	A1S_2037	NSD
WT *ddrR umuDAb*	KZA74_07480	NA	downreg
WT *ddrR umuDAb*	KZA74_07485	NA	downreg
WT *ddrR umuDAb*	KZA74_07510	NA	downreg
WT *ddrR umuDAb*	KZA74_07525	NA	downreg
WT *ddrR*	KZA74_07540	NA	NSD
WT *umuDAb*	KZA74_07545	NA	upreg
WT *ddrR umuDAb*	KZA74_07550	NA	NSD
WT *ddrR umuDAb*	KZA74_07555	NA	NSD
WT *ddrR umuDAb*	KZA74_07595	NA	downreg
WT *ddrR umuDAb*	KZA74_07600	NA	downreg
WT *ddrR umuDAb*	KZA74_07635	NA	downreg
WT *ddrR umuDAb*	KZA74_07650	NA	NSD
WT *ddrR umuDAb*	KZA74_07685	NA	NSD
WT *ddrR*	KZA74_07690	NA	downreg
WT *ddrR umuDAb*	KZA74_07710	NA	downreg
WT *ddrR umuDAb*	KZA74_07740	NA	downreg
WT	KZA74_07745	NA	upreg
WT *ddrR umuDAb*	KZA74_10255	NA	downreg
WT *ddrR umuDAb*	KZA74_10295	NA	downreg
WT *ddrR umuDAb*	KZA74_10325	NA	downreg
WT *ddrR umuDAb*	KZA74_10340	NA	downreg
WT *ddrR umuDAb*	KZA74_10370	NA	NSD
WT *ddrR umuDAb*	KZA74_10375	A1S_3690	NSD
WT *ddrR umuDAb*	KZA74_10385	A1S_3688	downreg
WT *ddrR umuDAb*	KZA74_10425	NA	downreg
WT *ddrR umuDAb*	KZA74_10430	NA	downreg
WT *ddrR umuDAb*	KZA74_10435	A1S_3686	downreg
WT *ddrR umuDAb*	KZA74_10445	A1S_1581	downreg
WT *ddrR umuDAb*	KZA74_10450	A1S_3684	downreg
WT *ddrR umuDAb*	KZA74_10455	A1S_3683	downreg
WT *ddrR umuDAb*	KZA74_10460	NA	downreg
WT	KZA74_12655	NA	NSD
WT *ddrR umuDAb*	KZA74_12680	NA	downreg
WT *ddrR umuDAb*	KZA74_12740	NA	downreg
WT *ddrR umuDAb*	KZA74_12810	NA	downreg
WT *ddrR umuDAb*	KZA74_12815	NA	upreg
WT *ddrR umuDAb*	KZA74_12885	NA	downreg
WT *ddrR umuDAb*	KZA74_12900	NA	downreg
WT *ddrR umuDAb*	KZA74_12905	NA	downreg
WT *ddrR umuDAb*	KZA74_12910	NA	downreg
WT *ddrR umuDAb*	KZA74_12915	NA	downreg
WT *ddrR umuDAb*	KZA74_12920	NA	downreg
WT *umuDAb*	KZA74_16395	A1S_0409	NSD
WT *umuDAb*	KZA74_18775*	A1S_0625	upreg

* Gene is encoded on pAB3.

NSD, No significant difference from WT expression.

NA, Not annotated as a coding region in ATCC 17978 genome assembly.

Shaded cells denote genes in cryptic prophage regions: orange = CP9; yellow = CP14; green = CP5.

Notably, we newly identified eight genes as being regulated by *ddrR* or *umuDAb*, five of which were not annotated in the ATCC 17978 genome assembly (**Table 3**). This brought the total number of induced genes controlled by these regulators to 23. Two coregulated genes (*KZA74_12655* and *KZA74_07745*) are potentially part of the *umuDC* operons they follow, and the other coregulated gene was the CP9-encoded putative c1 prophage repressor *esvI* (*KZA74_07470*) (**Figure 3C**). Three *ddrR*-regulated genes were new DEGs: *KZA74_18755*, *KZA74_16395* (located next to highly induced and conserved *gst* as described above), and *KZA74_07545* (the latter two had increased basal expression in the *ddrR* mutant). Two *umuDAb*-regulated genes were new DEGs in CP9: *KZA74_07540* and *KZA74_07690* (**Figure 3C**).

#### DNA damage induced many genes in *ddrR* and *umuDAb* mutant strains that are not induced in wildtype cells

We also identified 121 genes induced after DNA damage in the *ddrR* and *umuDAb* mutants that were not induced in WT cells (**Supplementary Table S6**). Fifty-five of these genes were only induced in the *ddrR* strain (**Figure 2B**), 33 of which were new to this study. Twenty-six genes were induced in both *umuDAb* and *ddrR* mutant strains, most of which (21) were not previously reported as induced (**Table 4**). These *ddrR*-regulated genes included five canonical DDR genes that were reported in a previous study (Hare et al., 2014) not to be induced: *uvrA* and *ruvA* (regulated by both *umuDAb* and *ddrR)*, and *ruvB*, *uvrC*, and *recX*, (regulated by *ddrR*). Forty genes were induced only in the *umuDAb* strain, including 11 ribosomal proteins and translation-related factors (**Table 5**).

**Table 4:** Newly identified genes only induced in *ddrR* and *umuDAb* mutants.

Induced in	17978 UN ENSEMBL GENE	ATCC 17978 A1S_ locus	*ddrR* basal Expression	*recA* basal Expression
*ddrR*	KZA74_00060	A1S_0012	downreg	NSD
*ddrR*	KZA74_00280	A1S_3427	NSD	NSD
*ddrR*	KZA74_00285	A1S_3426	downreg	NSD
*ddrR*	KZA74_00315	A1S_3421	downreg	NSD
*ddrR*	KZA74_00435	NA	downreg	NSD
*ddrR*	KZA74_00450	A1S_3392	upreg	NSD
*ddrR*	KZA74_00545	A1S_3372	downreg	NSD
*ddrR*	KZA74_01210	A1S_3252	downreg	NSD
*ddrR*	KZA74_01395	A1S_3219	downreg	NSD
*ddrR*	KZA74_01915	A1S_3114	downreg	NSD
*ddrR*	KZA74_07270	NA	downreg	NSD
*ddrR*	KZA74_07675	NA	NSD	NSD
*ddrR*	KZA74_09110	A1S_3726	downreg	NSD
*ddrR*	KZA74_09640	A1S_1707	NSD	NSD
*ddrR*	KZA74_10380	A1S_3689	NSD	NSD
*ddrR*	KZA74_10390	NA	downreg	NSD
*ddrR*	KZA74_10395	NA	downreg	NSD
*ddrR*	KZA74_10400	A1S_1585	downreg	NSD
*ddrR*	KZA74_11230	A1S_1433	downreg	NSD
*ddrR*	KZA74_11350	A1S_1410	downreg	NSD
*ddrR*	KZA74_14110	A1S_3576	downreg	NSD
*ddrR*	KZA74_17400	A1S_0203	downreg	NSD
*ddrR*	KZA74_17565	A1S_0171	downreg	NSD
*ddrR*	KZA74_17700	A1S_0142	NSD	NSD
*ddrR*	KZA74_18010	AS1_3486	downreg	NSD
*ddrR*	KZA74_18315	A1S_0024	NSD	NSD
*ddrR*	KZA74_18450	A1S_2965	upreg	NSD
*ddrR*	KZA74_18460	A1S_3870	downreg	NSD
*ddrR*	KZA74_18470	A1S_2969	NSD	NSD
*ddrR*	KZA74_18505	A1S_2976	upreg	NSD
*ddrR*	KZA74_18515	A1S_2978	upreg	NSD
*ddrR*	KZA74_18810*	A1S_3511	upreg	NSD
*ddrR*	repM**	NA	NSD	NSD
*ddrR and umuDAb*	KZA74_00625	A1S_3360	NSD	upreg
*ddrR and umuDAb*	*uvrA*	A1S_3295	NSD	NSD
*ddrR and umuDAb*	*ssb*	A1S_3287	NSD	NSD
*ddrR and umuDAb*	KZA74_01905	A1S_3116	NSD	upreg
*ddrR and umuDAb*	KZA74_01910	A1S_3115	NSD	NSD
*ddrR and umuDAb*	KZA74_07370	A1S_2051	upreg	NSD
*ddrR and umuDAb*	*murB*	A1S_2050	NSD	NSD
*ddrR and umuDAb*	*recA*	A1S_1962	NSD	NSD
*ddrR and umuDAb*	KZA74_09100	A1S_3727	NSD	NSD
*ddrR and umuDAb*	KZA74_10320	A1S_1587	downreg	NSD
*ddrR and umuDAb*	KZA74_10405	A1S_1584	downreg	NSD
*ddrR and umuDAb*	KZA74_10410	A1S_1583	downreg	NSD
*ddrR and umuDAb*	KZA74_10415	NA	downreg	NSD
*ddrR and umuDAb*	KZA74_10420	A1S_3687	NSD	NSD
*ddrR and umuDAb*	KZA74_10465	A1S_1580	downreg	NSD
*ddrR and umuDAb*	KZA74_12350	A1S_1226	downreg	NSD
*ddrR and umuDAb*	KZA74_12355	A1S_3630	downreg	NSD
*ddrR and umuDAb*	KZA74_12360	A1S_1225	NSD	NSD
*ddrR and umuDAb*	*parC*	A1S_0194	NSD	NSD
*ddrR and umuDAb*	KZA74_18475	A1S_3871	downreg	NSD
*ddrR and umuDAb*	KZA74_19055*	A1S_3536	downreg	NSD

NSD, No significant difference from WT expression.

NA, Not annotated as a coding region in ATCC 17978 genome assembly.

Shaded cells denote genes in cryptic prophage regions: orange = CP9; yellow = CP14.

* Gene is encoded on pAB3; ** Gene is encoded on pAB1.

**Table 5:** Newly identified genes only induced in *umuDAb* mutant.

17978 UN ENSEMBL GENE	ATCC 17978 A1S_ locus	*ddrR* Basal Expression	*recA* basal Expression
KZA74_00065	A1S_0013	NSD	NSD
*tyrS*	A1S_0014	upreg	NSD
KZA74_00100	A1S_3460	NSD	NSD
*ilvD*	A1S_3455	NSD	NSD
*aceE*	A1S_3328	NSD	NSD
*guaB*	A1S_3321	upreg	NSD
KZA74_00860	A1S_3314	NSD	NSD
KZA74_00985	A1S_3289	NSD	NSD
*rimM*	A1S_3163	upreg	NSD
*trmD*	A1S_3162	upreg	NSD
*rplS*	A1S_3161	upreg	NSD
*rplD*	A1S_3079	upreg	NSD
*rpsC*	A1S_3075	NSD	NSD
KZA74_07320	A1S_2061	upreg	NSD
*frr*	A1S_1974	upreg	downreg
*alaS*	A1S_1176	upreg	NSD
KZA74_12925	A1S_1142	upreg	NSD
KZA74_16540	A1S_0378	NSD	NSD
KZA74_17025	A1S_0279	upreg	NSD
*gpmI*	A1S_0230	upreg	NSD
*lldP*	A1S_0067	NSD	NSD
KZA74_18085	A1S_0066	NSD	NSD
*wbpD*	A1S_0054	NSD	NSD
*murJ*	A1S_0046	NSD	NSD
*ileS*	A1S_0020	NSD	NSD
*lspA*	A1S_0019	NSD	NSD
KZA74_18340	A1S_0018	upreg	NSD
KZA74_18375	A1S_r	downreg	NSD
*purE*	A1S_2964	upreg	NSD
*mpl*	A1S_2966	NSD	NSD
*yidC*	A1S_2980	upreg	NSD
*yidD*	A1S_2982	upreg	NSD
*rnpA*	A1S_2983	upreg	NSD
*rpmH*	A1S_2984	upreg	NSD
KZA74_18635*	A1S_0676	upreg	NSD
KZA74_18765*	A1S_0624	NSD	NSD
KZA74_19065*	NA	NSD	NSD
KZA74_19230*	A1S_0651	NSD	NSD
KZA74_01755	A1S_3146	Upreg	NSD
KZA74_18440	A1S_2963	Upreg	NSD

NSD = No significant difference from WT expression.

* Gene is encoded on pAB3.

More than half of the induced genes showed altered basal expression levels in the *ddrR* or *umuDAb* mutants. Twenty-nine genes induced in *ddrR* cells showed induction from basal levels significantly lower in *ddrR* than in WT, including *adeFGH, mgtA, mgtC, benE, czcC, sadH, gpi*, and *ltrA*. Seven other genes were induced from levels significantly *higher* in *ddrR* than in WT, including *pgpA (KZA74_00450), dgt* (*KZA74_04655*), a low molecular weight phosphotyrosine protein phosphatase (*KZA74_07370*), a metal/formaldehyde-sensitive transcriptional repressor (*KZA74_18515*), a DsbC family protein (*KZA74_18505*), a DMT family protein (*KZA74_18450*), and a hypothetical (*KZA74_18810*). Half of the forty genes induced only in the *umuDAb* mutant had basal expression levels that trended lower than WT, but this trend was not statistically significant. These mostly transcription/translation genes were, conversely, basally upregulated in the *ddrR* mutant.

### *ddrR* and *umuDAb* mutations significantly affect basal gene expression

**Figure 4:**
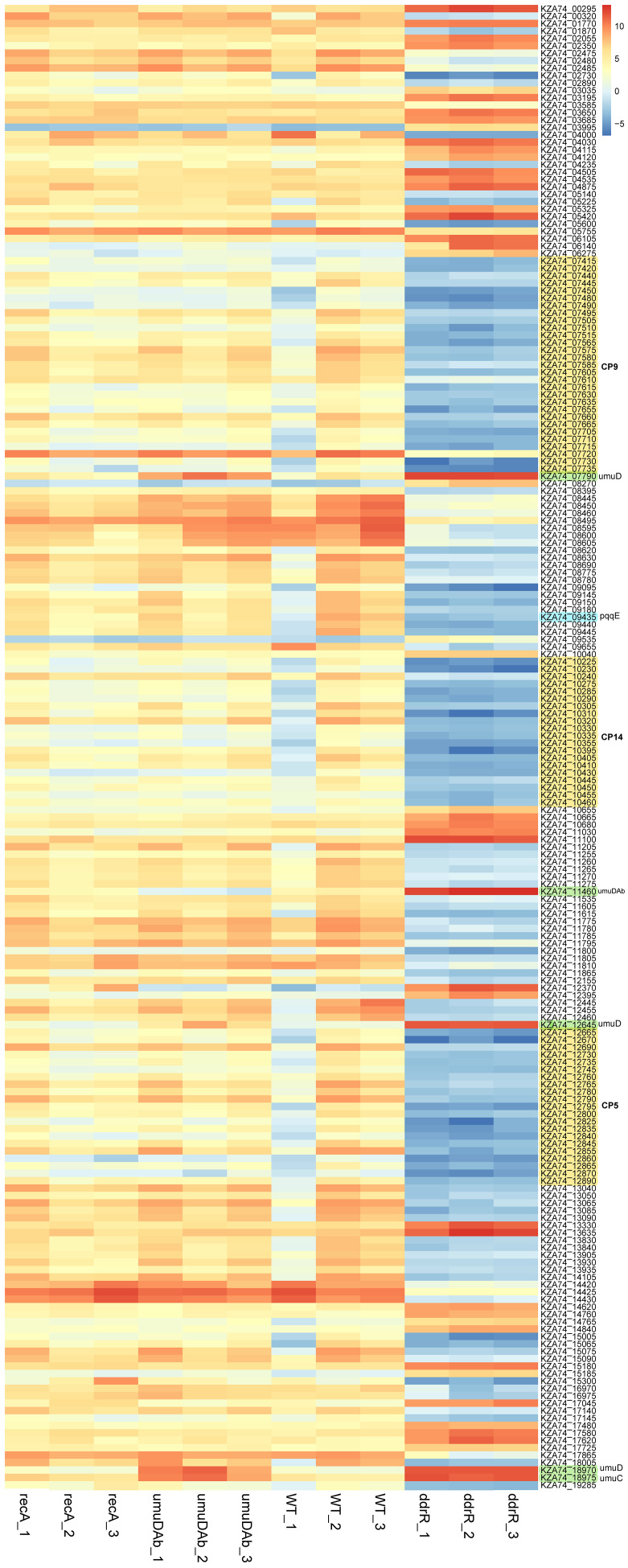
Heatmap of normalized read counts for the top 200 statistically significant DEGs (basal expression). Each column represents a biological replicate sample; each row represents a gene. Note the distinct and large-scale gene expression changes in the three clustered *ddrR* samples in the last three columns. The three cryptic prophages are highlighted in yellow and labelled, as are the *umuDC* and *umuDAb* loci. The log2 relative gene expression scale is depicted on the top right, with red representing upregulated and blue downregulated.

For basal expression comparisons we merged the untreated sample data for every strain’s growth in minimal media into a separate counts file to reduce any variance due to MMC treatment. A heatmap of the normalized read values for the top 200 differentially expressed genes was used to look for different expression patterns by strain. (**Figure 4**). The genes were classified as upregulated or downregulated for each untreated strain compared to the WT 17978 JH strain. The basal expression results for the mutant strains compared to the WT strain are in **Supplementary Table S7**.

#### Basal expression was much more affected by *ddrR* and *umuDAb* or *recA* mutation

**Figure 5:**
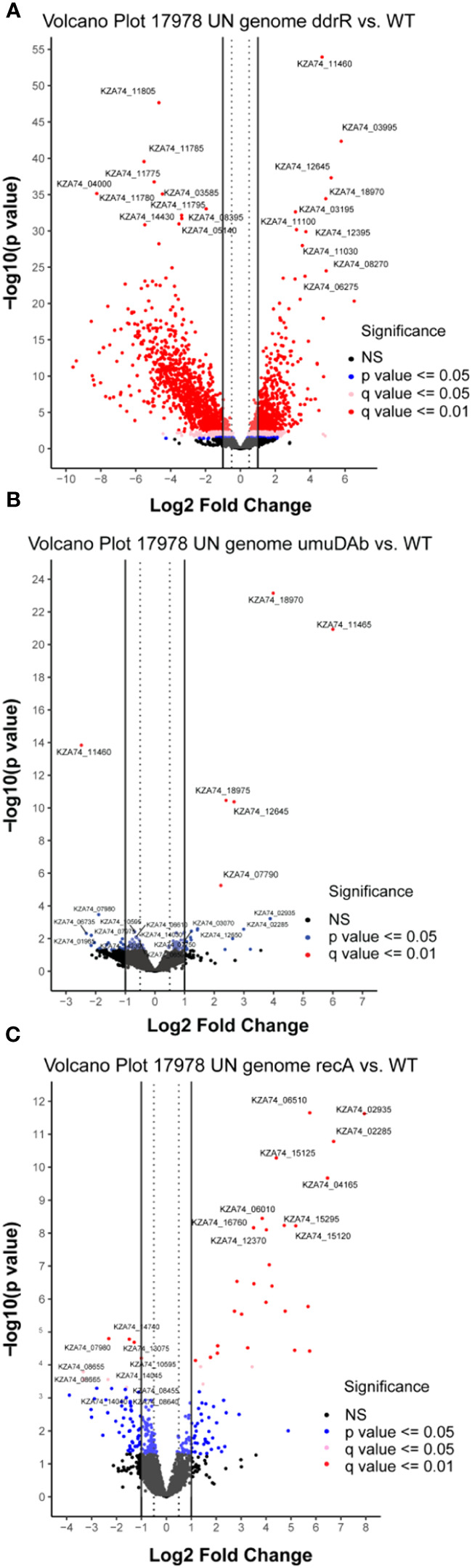
Global transcriptomic analysis of genes that were differentially expressed in WT *A. baumannii* 17978 JH cells vs mutants in **(A)** *ddrR*, **(B)** *umuDAb*, or **(C)** *recA*. Only untreated cells’ RNASeq data was combined and used in this analysis. Volcano plots show the log2 fold changes in gene expression where each dot represents one gene. Red and pink dots indicate significance at the q < 0.01 or q < 0.05 level. The log2 fold change is plotted on the x-axis, and the y-axis shows the log10 of the p value. The 10 genes with the highest induction or expression are labelled.

**Figure 6:**
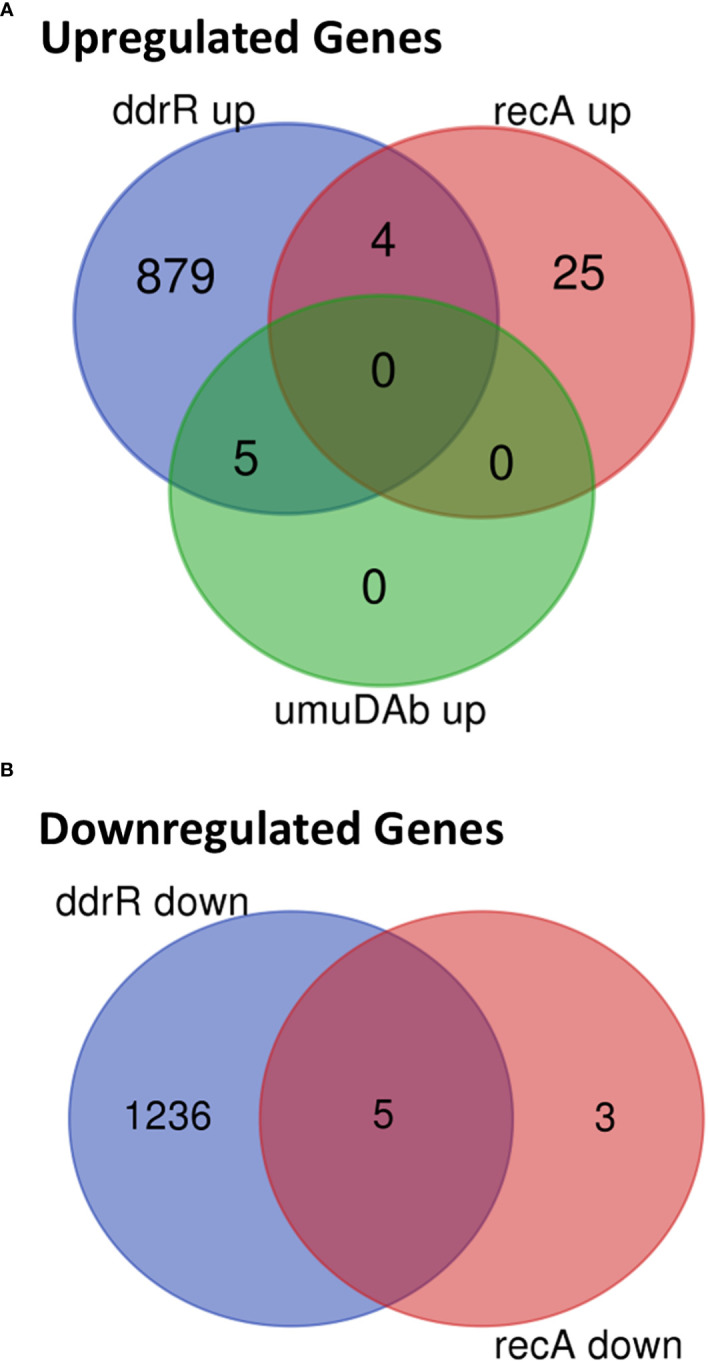
Venn diagram of differential gene expression, showing genes whose basal expression was **(A)** upregulated or **(B)** downregulated in WT *A. baumannii* 17978 JH cells vs *ddrR* and *umuDAb* mutants (q < 0.05 for each).

Mutation of *ddrR* tremendously impacted basal gene expression: 888 genes (23% of the genome) were significantly upregulated compared to WT (**Figures 5A**, **6A**, **SupplementaryTable S8**) and 1241 genes (32.1%) were downregulated compared to WT expression levels (**Figures 5A**, **6B**; **Supplementary Table S9**). Among the pathways enriched for upregulation were genes involved in cell division, DNA replication, protein transcription/translation/modification, phosphorylation, and non-acinetobactin iron acquisition. Also upregulated were three glutathione S-transferases (*gst*) and many genes involved in lipid metabolism, LPS biosynthesis, lysine biosynthesis, methionine import, pH regulation, phosphate import, phosphorylation, protein biosynthesis, OMP folding and transport, purine synthesis and metabolism, signal transduction, and sulfur metabolism. Notably, at least eleven transcriptional repressors and 72 transcription/translation genes (from ribosome proteins and assembly to extension factors and terminators) were also upregulated.

**Figure 7:**
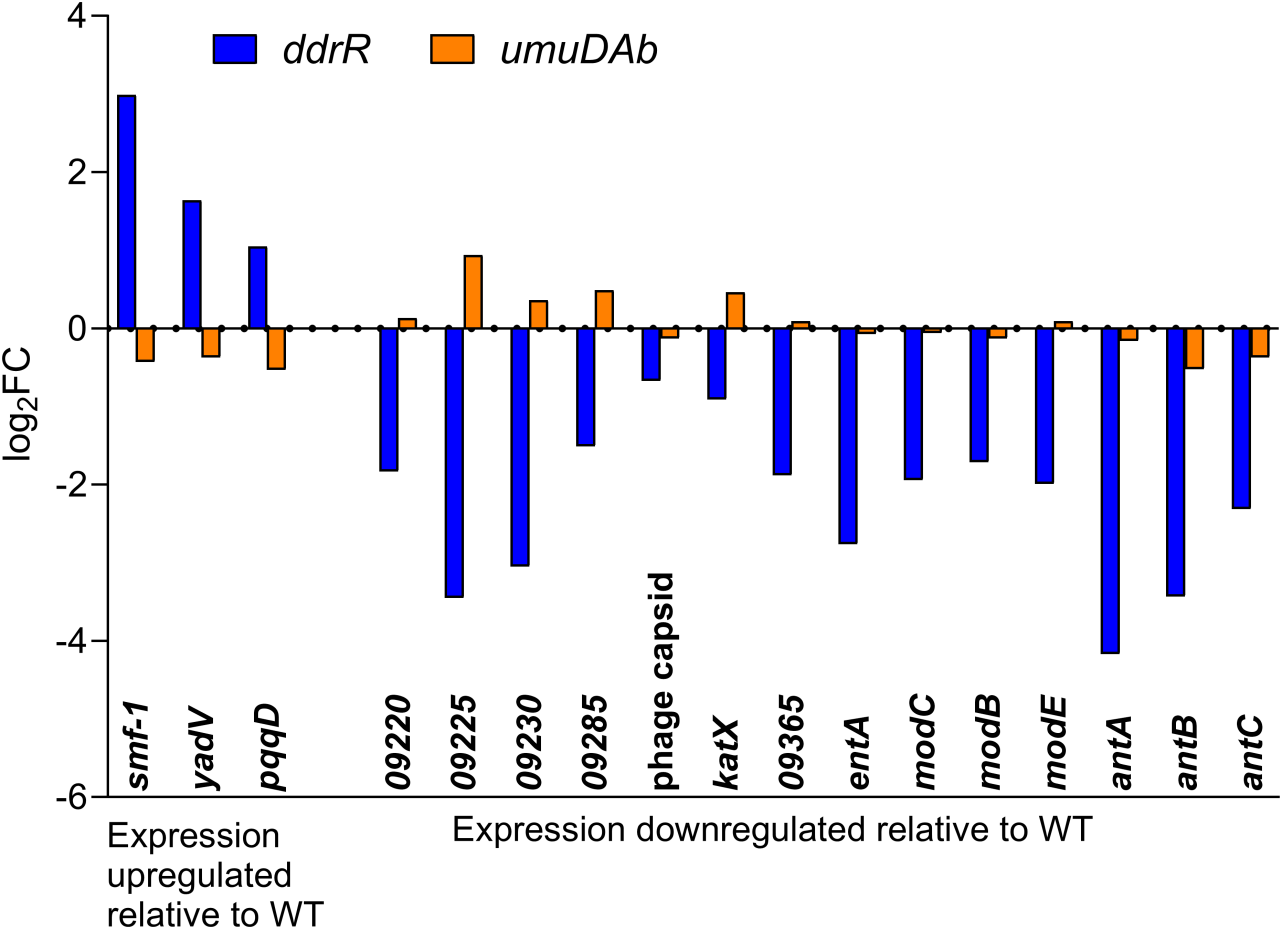
AbaAL44 genes displaying differential expression in the absence of MMC treatment in *ddrR* and *umuDAb* mutant strains. RNASeq data of log_2_ fold changes is shown for all 17 genes that were basally upregulated (n = 3) or downregulated (n = 14) in the *ddrR* strain.

**Figure 8:**
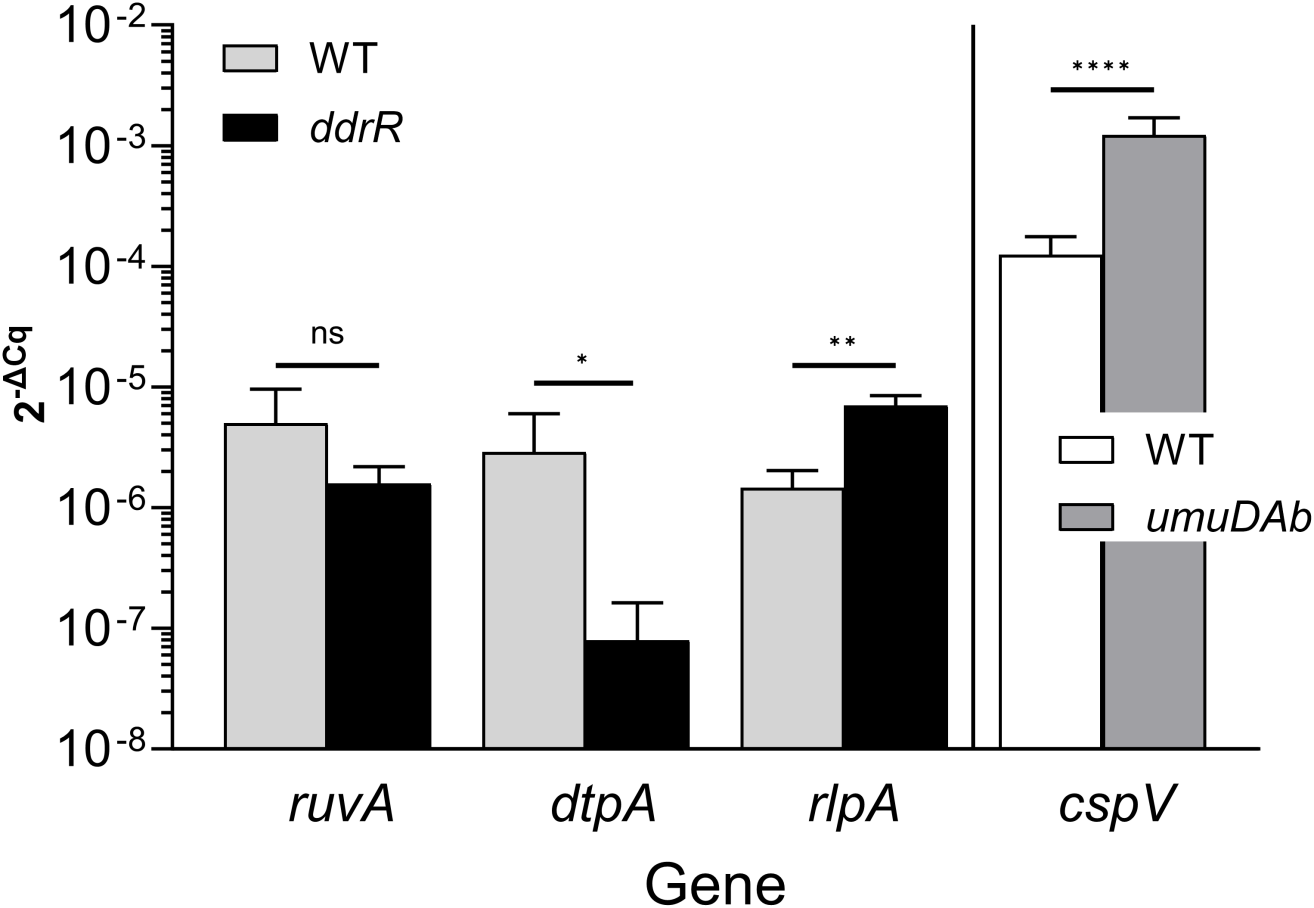
Representative RT-qPCR validation. This figure shows relative expression data for genes *ruvA, dtpA, rlpA*, and *cspV* in WT, *ddrR*, and/or *umuDAb* strains. RT-qPCR experiments measured gene expression (2^-ΔCq^) in the absence of DNA damage (basal expression). The expression of genes from different regulation categories is shown: genes whose expression was unchanged in *ddrR* and *umuDAb* mutant strains relative to WT (*ruvA; KZA74_04650*); those whose expression was decreased in the *ddrR* mutant relative to WT (*dtpA*; *KZA74_11470*); those whose expression was increased in the *ddrR* mutant relative to WT (*rlpA*; *KZA74_05975*); and those whose expression was increased in the *umuDAb* mutant relative to WT (*cspV*; *KZA74_12340*). The standard deviation of the mean from technical triplicates of biological triplicates is shown, where results from t-tests are shown: ****,P < 0.0001; ***,P < 0.001; **,P < 0.01; *,P < 0.05; ns = not significant.

Two AbaAL44 genes, encoding a type 1 fimbrial protein (*smf-1*) and chaperone *yadV* were upregulated (**Supplementary Table S4**; **Figure 7**). Other chromosomal upregulated genes were involved in cell division processes that facilitate cellular elongation, septal formation, and daughter cell separation as part of the normal cell cycle. For example, the *tol-pal* system is a required part of cell division (Yakhnina and Bernhardt, 2020; Hale et al., 2022; Park and Cho, 2022). In the *ddrR* mutant, the *tol-pal* operon had a higher basal expression when compared to WT. Also upregulated was the newly characterized gene *advA* involved in chromosome segregation and cell division in *A. baumannii* (Geisinger et al., 2020) and t he lytic transglycosylase *rlpA* which facilitates daughter cell separation in *Pseudomonas aeruginosa* (Jorgenson et al., 2014) and *Vibrio cholerae* (Weaver et al., 2019). This upregulation of *rlpA* was confirmed with RT-qPCR analyses (**Figure 8**). In the *ddrR* mutant, *rlpA* and other genes mentioned in those studies (*mltB* and *mltG*) were all upregulated while amidase, *amiE*, and a lytic transglycosylase, *slt*, were downregulated.

The lack of *ddrR* also affected expression of stringent response genes. The stringent response can be triggered by production of the alarmones guanosine tetraphosphate (ppGpp) and guanosine pentaphosphate (pppGpp) in response to nutrient deprivation (Liu et al., 2017). The gene *relA* (*KZA74_15545*) is responsible for the ppGpp synthesis and accumulation of ppGpp, and *spoT* regulates ppGpp accumulation with hydrolytic activity and weak synthetase ability (Gaca et al., 2015). In *A. baumannii*, ppGpp deficiency can affect biofilm formation (Kim et al., 2021) and efflux pump expression (Jung et al., 2020). We observed s*poT* (*KAZ74_01610*) to be upregulated 3.4-fold in the *ddrR* mutant.

**Figure 9:**
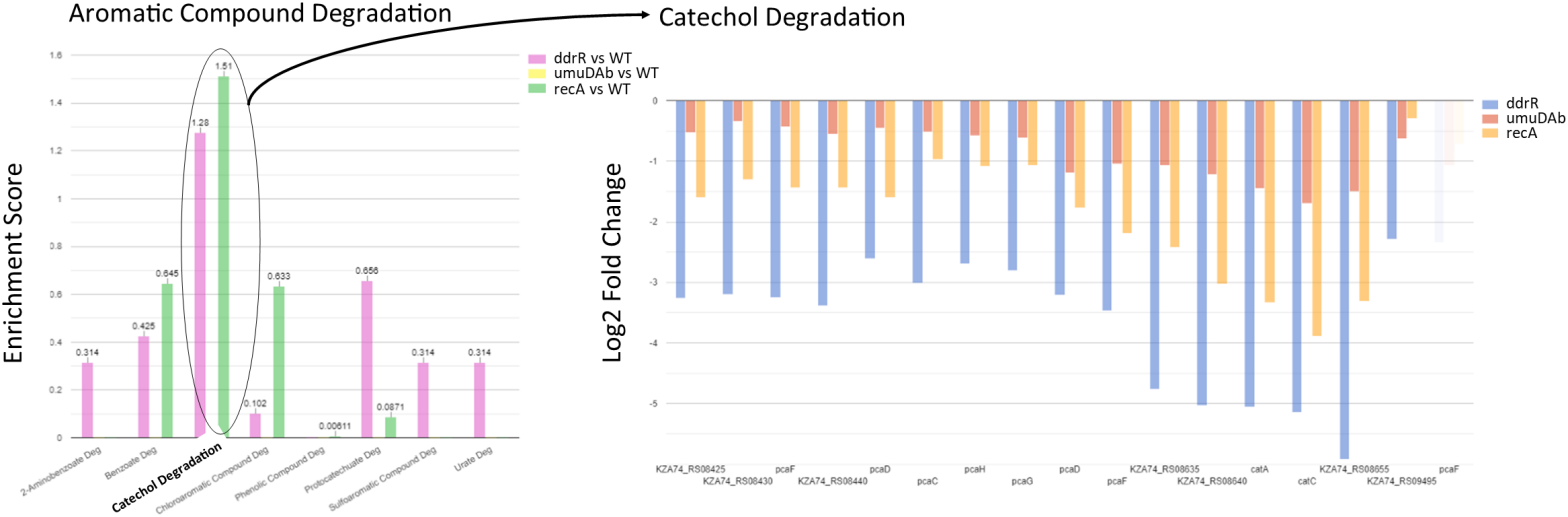
Example of pathway enrichment analysis. This figure shows a sample enrichment analysis for some genes whose basal expression was downregulated in the *ddrR* mutant. Multiple pathways were enriched for aromatic compounds. The *ddrR* cells were enriched for 16 of the 17 known genes involved in catechol degradation.

Genes downregulated in the *ddrR* mutant included 14 of the 38 genes in AbaAL44 (**Figure 7**, **Supplementary Table S4**), 80% of the prophage genes (146 out of 179 genes) and at least 71 transcriptional regulators/repressors. They also included at least 20 amino acid transporters and 23 ABC and MFS transporters. Pathway analysis showed that these downregulated genes are involved in acyclic terpene and leucine/isovalerate utilization, aromatic catabolism, amine metabolism, benzoate metabolism (20 genes), carnitine metabolism, catechol metabolism (for example analysis, see **Figure 9**), cell adhesion, fimbrial biogenesis, type II, IV, and VI secretion systems, cytolysis, fatty acid metabolism, glycerol metabolism, magnesium transport, nitrite metabolism, potassium transport, leucine catabolism, muconate metabolism, organosulfur metabolism, phenylalanine, tyrosine and tryptophan biosynthesis, purine metabolism, thiamine metabolism, isoleucine and valine catabolism, vanillin metabolism, acinetobactin-mediated iron acquisition, and copper and zinc regulation (**Supplementary Table S9**). Specific downregulated genes were the *csuBABCDE* biofilm genes, fimbrial genes; *fimT* and *fimB*, pilus genes; *pilV, pilGHIJ, pilT, pilU, pilBC, pilR, pilNOPQ*, and *pilA;* and efflux pump genes *adeRS, adeFGH*, and *adeL*. Of note are two separate clusters of oxidative and desiccation stress-related genes found downstream of *ddrR* in 17978 JH, all of which are downregulated in the *ddrR* mutant strain (Sun et al., 2021).

The significant amount of dysregulation seen in the *ddrR* mutant was not shared by the *umuDAb* or *recA* mutants. In the *umuDAb* mutant, basal expression was significantly higher than in WT cells for only five genes: the four error-prone polymerase V genes (three *umuD* and one *umu*C allele), and *ddrR* (Hare et al., 2014) (**Figures 5B**, **6A**; **Supplementary Table S8**). These five genes are repressed by *umuDAb* and *ddrR* and induced after DNA damage (Hare et al., 2014; Peterson et al., 2020). No genes that showed decreased expression in the *umuDAb* mutant strain were statistically significant.

The *recA* mutant had a relatively small number of genes (37) differentially expressed. Eight were downregulated (**Supplementary Table S9**), and 29 were upregulated (**Figures 5C**, **6**, **Supplementary Table S8**). Interestingly, several genes were similarly dysregulated in multiple mutants. Among upregulated genes, four were shared between the *recA* and *ddrR* mutants and five were shared between the *umuDAb* and *ddrR* mutants. The *recA* and *ddrR* mutants also shared five downregulated genes (**Figure 6**).

#### Essential genes’ basal expression was dysregulated in the *ddrR* mutant and downregulated in the *umuDAb* mutant

Several studies have determined which genes are essential in *A. baumannii* (Wang et al., 2014; Gallagher et al., 2015; Uddin et al., 2019; Bai et al., 2021). The most recent study found 373 genes considered essential (Bai et al., 2021). We considered a gene essential if it was on this list, as it compared their data to previous studies.

Many differences were seen in essential gene expression between our mutant strains and WT when grown under minimal media conditions. More than half (189) of these essential genes were expressed significantly higher in the *ddrR* mutant than in the WT strain (**Supplementary Table S10**). These essential genes are involved in amino acid (AA)-tRNA synthetases, ATP synthase and purine synthesis, capsular polysaccharide synthesis (CPS), DNA, replication, dNTP synthesis, fatty acid synthesis, Fe-S cluster synthesis, gluconeogenesis, heme synthesis, histidine synthesis, isoprenoid synthesis, lipopolysaccharide (LPS) synthesis, peptidoglycan (PG) synthesis, mRNA degradation, methionine synthesis, nicotinamide synthesis, phospholipid synthesis, protein secretion, purine synthesis, pyrimidine synthesis, ribosomal synthesis, ribosomal modification, ribosomal proteins, sodium efflux, transcription, transcription regulation, translation factors, tRNA modification, and tryptophan synthesis.

Second, many (85) of these 189 genes were inversely regulated in the *ddrR* and *umuDAb* mutant strains, with genes upregulated in the *ddrR* mutant being downregulated in the *umuDAb* mutant, often more than two-fold lower than WT levels, although the p-adjusted values were not significant. Several of the lower-expressed genes in the *umuDAb* mutant that code for ribosomal, transcription and translation proteins had expression levels increased more than 20-fold after DNA-damage, to the level of WT expression. Since these are essential genes, the reduced levels in the *umuDAb* mutant are likely biologically significant.

Conversely, only five essential genes were expressed significantly lower in the *ddrR* mutant than the WT strain. Two of these encode hypothetical proteins *KZA74_09110* and *KZA74_11475*, which is transcribed divergently from *dtpA and* was not annotated in the ATCC 17978 genome. Both were induced after DNA damage in the *ddrR* mutant. The three other genes, *prtC* (*KZA74_13285*), *hfq* (*KZA74_07030*), and *hemD* (*KZA74_17100*) were downregulated in the *ddrR* mutant and unchanged after MMC treatment in all strains.

## Discussion

For this project we combined the use of the new reference genome, 17978 UN, that more closely matched our lab strain (Wijers et al., 2021), with an improved analysis pipeline that included DESeq2. The first widely used *A. baumannii* genome assembly and annotation (ATCC 17978) had all pAB3 genes combined with the chromosome. While it is possible that pAB3 may have integrated into the chromosome in the ATCC 17978 strain used for the genome assembly, others have observed no evidence of integration in their study of pAB3 (Weber et al., 2015). As the location and nature of gene acquisition matters for understanding the nature of any regulon, it is important to use an accurate genome assembly (with correct contig assembly and gene annotations).

Earlier analyses investigated genes controlled by the unusual UmuDAb and DdrR coregulators and focused on the genes induced after DNA damage (Aranda et al., 2013; Hare et al., 2014; Peterson et al., 2020). Those previous studies did not characterize genes repressed after DNA damage and genes that rely on *ddrR* or *umuDAb* for their repression or induction but are not part of the DDR. In this work, we showed how aligning our RNASeq data to the improved 17978 UN genome assembly for *A. baumannii* revealed 43 additional genes that were responsive to DNA damage, one-third of which were regulated by either *ddrR* or *umuDAb*. We expanded our knowledge of the control of the DDR, in observing that 100% of the 12 genes newly identified as repressed were regulated by either *umuDAb* or *ddrR* but only 17% of the 48 genes newly identified as induced after DNA damage were regulated by *umuDAb* or *ddrR*. Furthermore, we identified newly annotated genes such as the hypothetical genes *KZA74_12655* and *KZA74_07745* that appear to be part of the two *umuDC* operons possessed by 17978 JH. Their possible role in the unusual DDR of this genus is unexplored and highlights that *ddrR* and *umuDAb* control the repression of genes induced after DNA damage.

The induction of the five canonical SOS genes *uvrA* and *ruvA*, *ruvB*, *uvrC*, and *recX* in the mutants suggests that *umuDAb* and *ddrR* might prevent these damage response genes from being induced after MMC treatment, which could potentially allow more error-prone DNA repair to happen. This additional control is an example of distinctive features in *A. baumannii* strains that have the potential to increase genomic variation.

DEGs in the newly identified AbaAL44 accessory region (Wijers et al., 2021) revealed no genes that were induced after DNA damage in WT cells, but three AbaAL44 genes were repressed. One of these genes was not regulated by *umuDAb* or *ddrR*, another uniquely required *ddrR* for repression, and one uniquely required *umuDAb*. Almost half of the genes in AbaAL44 (17) were basally dysregulated in the absence of *ddrR*, indicating its importance in regulating genes in this pathogenicity island. These controlled genes encode a zonular occludens toxin domain protein (*KZA74_09220*), type 1 fimbrial protein and chaperone, and all three genes of the *antABC* operon (anthranilate dioxygenase) (**Supplementary Table S4**).

Furthermore, we reveal a large regulatory role for *ddrR* in controlling the expression of hundreds of genes in the absence of DNA damage. Quorum sensing (QS) is a form of cell-cell communication that allows pathogenic bacteria to coordinate virulence gene expression. QS is regulated in *A. baumannii* by the *abaIMR* operon (López-Martín et al., 2021; Sun et al., 2021), a gene cluster evolutionarily conserved among many *Acinetobacter* species The gene *abaI* encodes acyl homoserine lactone synthase, involved in signal transduction and potentially enhances *A. baumannii* virulence. AbaR is an AI synthase receptor. AbaM represses AHL biosynthesis and is a regulatory component of many other genes involved in QS and independent of QS. The genes *abaI* and *abaR* are upregulated in an *abaM*::TN26 mutant (López-Martín et al., 2021). In the *ddrR* mutant all three of these genes were downregulated.

Biofilms and fimbrial biogenesis are ways bacteria protect themselves from harsh living environments and biofilms have multiple avenues of regulation, such as cellular ppGpp levels. Many of the genes that are responsible for biofilm production, efflux pumps, motility, capsule formation, and siderophore production affected by ppGpp depletion (Kim et al., 2021), were downregulated in the *ddrR* mutant. Inversely, many essential genes are upregulated in the absence of *ddrR* and downregulated in the absence of *umuDAb*. None of the genes downregulated in the *umuDAb* mutant were statistically significant, but as essential genes, decreases in their expression are biologically significant as shown by CRISPR-based gene knockdown experiments by Bai et al., 2021. Without UmuDAb to regulate *ddrR* production, excess *ddrR* product could inhibit these genes.

The large number of genes dysregulated in the *ddrR* mutant was unexpected (1241 downregulated and 888 upregulated). One issue that can limit the confidence in comparisons of datasets is batch effects, due to the *ddrR* mutant samples being sequenced at a different time with paired-end reads rather than the single-end sequences used for all the other samples. Even though the same scientist used the same lab equipment and growth conditions to collect these samples, we examined our data in additional ways to address this concern. First, wondering if the adjusted p-value cutoff of 0.05 was too lenient, we reran the analysis with a more stringent cutoff of padj < 0.01 and adding a log2 fold change cutoff greater than 1 and less than -1. This method still yielded 82.8% (1028 genes) of the genes obtained with the higher p-value, and the number of basally unregulated genes only decreased to 59.9% (532 genes) of the number obtained with the higher p-value. Second, several genes were selected from those mentioned in this study for validation with RT-qPCR and several others had already been validated in previous work (see summary in **Table 6**). The results were not concordant for 0% of the DNA damage-responsive genes and only 20% of the genes whose basal expression was dysregulated. This was not surprising as the results for 15-20% of genes, when compared between RNAseq and RT-qPCR, can be non-concordant (not matching between methods) (Everaert et al., 2017; Coenye, 2021). However, it has been noted that in studies with large numbers of DEGs, if the RNAseq analyses pipeline is robust enough and the sample number is high enough there is not much value added with qPCR validation (Coenye, 2021). The DESeq2 pipeline used here is robust and can detect and reduce batch effect in its results (Love et al., 2014; Seyednasrollah et al., 2015). Even though all of the genes tested were not concordant, the validation of the majority of the genes selected for RT-qPCR lends credence to the conclusion that the product of *ddrR* affects many cellular systems and processes. Finally, we chose the DESeq2 algorithm that uses a statistical model to estimate the mean and variance of the count data, which controls for batch effects, such as our differences between sequencing methods (the single and paired-end reads). Crucially, this approach allowed us to compare the expression of DNA damage-responsive genes and other genes directly and comprehensively across WT and all the mutant strains without DNA damage. DESeq2 has good sensitivity for detecting low-expressed genes and is known for more consistent detections in cases with few biological replicates (Love et al., 2014; Seyednasrollah et al., 2015), as we had biological triplicates for each of our strains. Nevertheless, the effects of the DdrR and UmuDAb regulators on any particular operon, metabolic process or gene will require additional investigation to confirm.

**Table 6:** Genes verified by RT-qPCR to match the pattern of regulation observed in RNASeq results.

Regulation pattern	Genes verified	Genes not verified	Reference
Induced after MMC treatment in WT but not in *ddrR* or *umuDAb*	umuD 18970*, *umuC 18975, umuD 12645, umuC 12650, umuC 07750, umuC 07790*, *ddrR*, *umuDAb*		(Hare et al., 2014; Peterson et al., 2020)
Repressed after MMC treatment in WT but not in *ddrR or umuDAb*	*lpdA*, *rlpA*, *ybfU*		This study
Repressed after MMC treatment in WT but not in *umuDAb*	*cspV*		This study
Repressed in *ddrR* but not WT	*copC*		This study
			
Basal expression unchanged in *ddrR*	*esvI*, *ssb*, *xerC*, *gst*, *ruvA*, *parE*		This study, (Hare et al., 2014; Peterson et al., 2020)
Basal expression increased in *ddrR*	*rlpA*, *umuD 18970*, *umuC 18975, umuD 12645, ddrR*, *umuDAb*	*scpA*, *yfbU, advA*	This study
Basal expression decreased in *ddrR*	*dtpA*	*benA*, *esvK* *lpdA*	This study
Basal expression increased in *umuDAb*	*cspV, umuD 18970*, *umuC 18975, umuD 12645, ddrR*, *umuDAb*		This study

*Numbers represent the KZA74 gene number where multiple genes have the same name.

Although it affects the expression of many genes, the mechanism of action of *ddrR* has not yet been elucidated. It co-regulates the error-prone polymerases, *umuDAb* and itself (Peterson et al., 2020) by enhancing the repression activity of the LexA-like repressor, UmuDAb (Cook et al., 2022), and DdrR has been shown to interact with UmuDAb protein *in vitro* (Pavlin et al., 2022). DdrR actions resemble in some ways those of the small bacteriophage protein, gp7, found in *Bacillus thuringiensis*, which binds to the bacterial LexA protein and increases its repression (Fornelos et al., 2015). However, DdrR is not prophage-encoded, shares no sequence similarity with gp7 (Cook et al., 2022; Pavlin et al., 2022), and interacts with the non-canonical UmuDAb repressor, not LexA. Nevertheless, its corepressor activities allow speculation that it may interact with other repressors or activators to modulate their regulatory activity. Alternately, DdrR is required for the induction of phage repressors *KZA74_07470* (*esvI*) and *KZA74_12875*, which may control multiple phage genes. Further studies and testing are still needed to reveal how *ddrR* interacts and affects other gene expression in *A. baumannii*.

This study pinpointed canonical damage response genes induced after DNA damage solely in the absence of *ddrR* or *umuDAb*, suggesting their potential role in preventing the induction of those genes and influencing genomic variation in *A. baumannii* strains. Moreover, the lack of *ddrR* led to the upregulation of essential genes involved in DNA replication, transcription, translation, and membrane transport, alongside alterations in stringent response pathways indicating a broader impact on cellular functions beyond DDR. The findings highlighted intricate regulatory networks affected by *ddrR*, particularly in biofilm formation, quorum sensing, and cellular communication, with implications for *A. baumannii* virulence and survival strategies. Future investigations aim to elucidate *ddrR*’s functional mechanisms, potentially paving the way for therapeutic targeting in combating *A. baumannii* infections.

## Data availability statement

Publicly available datasets were analyzed in this study. This data can be found here: SRA run selector: SRR1165107 SRR1165108 SRR1165109 SRR1165110 SRR1165111 SRR1165112 SRR1165113 SRR1165114 SRR1165115 SRR1165116 SRR1165117 SRR1165118 SRR1165119 SRR1165120 SRR1165121 SRR1165122 SRR1165123 SRR1165124 SRR6150760 SRR6150759 SRR6150758 SRR6150757 SRR6150756 SRR6150755.

## Author contributions

DC: Conceptualization, Data curation, Formal Analysis, Investigation, Methodology, Software, Validation, Visualization, Writing – original draft, Writing – review & editing. MF: Investigation, Visualization, Writing – review & editing. JC: Validation, Writing – review & editing, Formal Analysis, Methodology. JH: Conceptualization, Funding acquisition, Investigation, Supervision, Validation, Visualization, Writing – review & editing.
