# Supplementary material for: DNA damage response coregulator ddrR affects many cellular pathways and processes in Acinetobacter baumannii 17978
Source: Front Cell Infect Microbiol. 2024 Jan 11;13:1324091. doi: 10.3389/fcimb.2023.1324091 (PMC10808703; doi:10.3389/fcimb.2023.1324091)
Supplement: Supplementary file 1 [file DataSheet_1.docx]

Tables S3-9. Colored cells in columns B through K designate prophage genes for the three cryptic prophage (CP) regions and the AbaAL44 region in *Acinetobacter baumannii* 17978; CP9 = Orange background with dark blue text, CP14 = yellow background with brown text, CP5 = Green background with dark green text, AbaAL44 = Light yellow background with black text. Colored text is only used for genomic molecule designations: the chromosome (black text), pAB1 (orange text), pAB2 (dark blue text), and pAB3 (green text).
