# Supplementary material for: DNA damage response coregulator ddrR affects many cellular pathways and processes in Acinetobacter baumannii 17978
Source: Front Cell Infect Microbiol. 2024 Jan 11;13:1324091. doi: 10.3389/fcimb.2023.1324091 (PMC10808703; doi:10.3389/fcimb.2023.1324091)
Supplement: Supplementary file 2 [file Table_1.docx]

Table S1. Primers used to verify regulation in RT-qPCR experiments.

| **Gene target** | **Sequence** | **Reference** |
| --- | --- | --- |
| *advA* | AAGAAGCTGCTCAACGTTTAG  GTTAGGCATTGCAACAAGCTG | This study |
| *benA* | TCAAAGCCATAGGAGCCACC  TGGTGCCGATGGTTATCACG | (Peterson et al.) |
| *copC* | CTACGCAATGTATTAGTTGGTGC  GGGTTGACTTGCTACAGAAGC | This study |
| *cspV* | TCTGAGATTCAAACACAAGGC  TGATATTCGAAGCTTGTGGG | This study |
| *ddrR* | AAACGGAAATGGTAATTTGAAGAG  TCATCTGCATAACCTAAAACTGC | (Peterson et al.) |
| *dtpA* | GTTCAGGTACTTCAAATCGTG  GCCTCTTCTGAGGTAAATTCA | This study |
| *esvI* | GAGTGGCTCCCTTTACCTGA  ATCGCCAGATTGCATGTCAC | (Hare et al.) |
| *esvK* | CAATTGGGGTTCTGGTCGTG  ATCTGTTCCACCTCTTGCCA | (Hare et al.) |
| *gst* | TGGGAAAGTGCCTGTATTGGT  TTCGGATCATCTAAGCAGGTGC | (Peterson et al.) |
| *hemP* | CACCTTTTAGCTTATTTACACGC  CGTAAACGATATTCCTCGCC | This study |
| *lpdA* | CGGACAGTTTGGTTTTCAGG  AGCGCGACCACTAAAAACAG | This study |
| *parE* | GCATGCAGCTTGGTTAAAATG  CGATGAAGCCTTAGCTGGTC | This study |
| *rlpA* | AACGTTCTGGCGATCAAATC  TTGACGACCATACCACGATG | This study |
| *ruvA* | TTTGGTTAAAGTTCCGGGGG  CAAATTGAATCTGAGGCAGGG | This study |
| *scpA* | TTACACGATGCTTTGTTCTGTG  CGTCGCCTGACTCTAATTTCT | This study |
| *ssb* | ATAACAACCAAGGTGGGGGC  GGTTGTTGTGGCGCTTTAGG | (Hare et al.) |
| *umuDAb* | TACCACATTCCTTTGGCGAC  TCCGGCATCTAACATGGACA | (Hare et al.) |
| *umuD 18970* | AAGTTGTTGCTCGGTCTCGG  TGCCGGTGATGGAAATCCTG | (Peterson et al.) |
| *umuC 18975* | TCTGAATCAATGCGCTCAAGACA  TCAAGTGCGGATAGGTTTTTGCT | (Peterson et al.) |
| *umuD 12645* | CTCTCGACATGAACGAGCAC  CCGTGTTTGGCATCAAGACT | (Hare et al.) |
| *umuC 12650* | CGTTTCTGAAGTTTGGGGCG  CGTAGATCGTGCGAGCCATA | (Peterson et al.) |
| *xerC* | AACTGGTTTTTCAGGCATGG  TTATGAAGTGGGCTGAACAGG | This study |
| *yfbU* | TGCCCTTTATGAAAACCTTCC  AGTTCTGGCTCACGATTTCC | This study |
